# Point/counterpoint: Cerebrovascular resistance is a flawed concept

**DOI:** 10.1177/0271678X231172854

**Published:** 2023-04-27

**Authors:** Jonathan Ince, Jatinder S Minhas, Ronney B Panerai

**Affiliations:** 1 Cerebral Haemodynamics in Ageing and Stroke Medicine (CHiASM) Group, Department of Cardiovascular Sciences, 4488 University of Leicester, Leicester, UK; 2 NIHR Leicester Biomedical Research Centre, Leicester, UK

**Keywords:** Cerebrovascular resistance, cerebral vascular conductance, critical closing pressure, cerebral autoregulation, resistance-area product

## Abstract

The relationship between cerebral blood flow and blood pressure is a critical part of investigation of cerebral autoregulation. Conventionally, cerebrovascular resistance (CVR) has been used to describe this relationship, but the underlying principles used for this method is flawed in real-world application for several reasons. Despite this, the use of CVR remains entrenched within current literature. This ‘Point/Counterpoint’ review provides a summary of the flaws in using CVR and explains the benefits of calculating the more accurate critical closing pressure (CrCP) and resistance-area product (RAP) parameters, with support of real-world data.

Cerebral autoregulation (CA) is the homeostatic mechanism of the brain which regulates cerebral blood flow (CBF) to variations in cerebral perfusion pressure (CPP). The assessment of CA plays a pivotal role in the understanding of the physiology of the brain, and in turn the effects that disease has on these processes. The optimal approach to assessing and describing CA has long been discussed, with many studies opting for the use of cerebrovascular resistance (CVR) as a measure of interest.

CVR is a measure of the vasomotor properties of blood vessels supplying the brain, and is described as a ratio between CPP and CBF, with CPP usually defined as the difference between arterial blood pressure (BP) and intracranial pressure (ICP). In some cases, due to the non-linearity of this relationship, the ratio can be reported as CVR = ΔCPP/ΔCBF. In situations where cerebral blood velocity (CBv) is measured using transcranial Doppler to estimate CBF, CVR can be expressed as an ‘index’ (CVR_i_).

Whilst CVR may appear as an attractive measure to describe CA, the physical concept of CVR follows Poiseuille’s law, which cannot be applied strictly to the cerebral circulation. Poiseuille’s law describes a proportional relationship between flow and the pressure drop along a single vessel, which can be valid for a rigid cylindrical tube within certain conditions (such as laminar flow), however the microcirculation (which represents the largest segmental resistance to blood flow) does not meet these conditions.

Two main characteristics of small cerebral vessels make CVR a misleading parameter. Firstly, small vessels such as arterioles and capillaries are collapsible, giving way to the phenomenon of vascular waterfall.^1^ Second, vascular smooth muscle and pericytes generate vascular wall tension, increasing the propensity of small vessels to collapse, giving rise to the phenomenon of the critical closing pressure (CrCP).^2^

The presence of CrCP makes supporting CVR challenging. With CrCP, there is an implication that CBF will stop at BP values greater than zero, however CVR (or CVR_i_) assumes that CBF (or CBv) will reach zero only when BP = 0 (Figure 1). A relatively large body of literature demonstrates the presence of CrCP in the cerebral circulation, with CrCP values as high as 50 mmHg, even in healthy subjects, and only occasionally can it be assumed to be near zero.^3^

**Figure 1.**
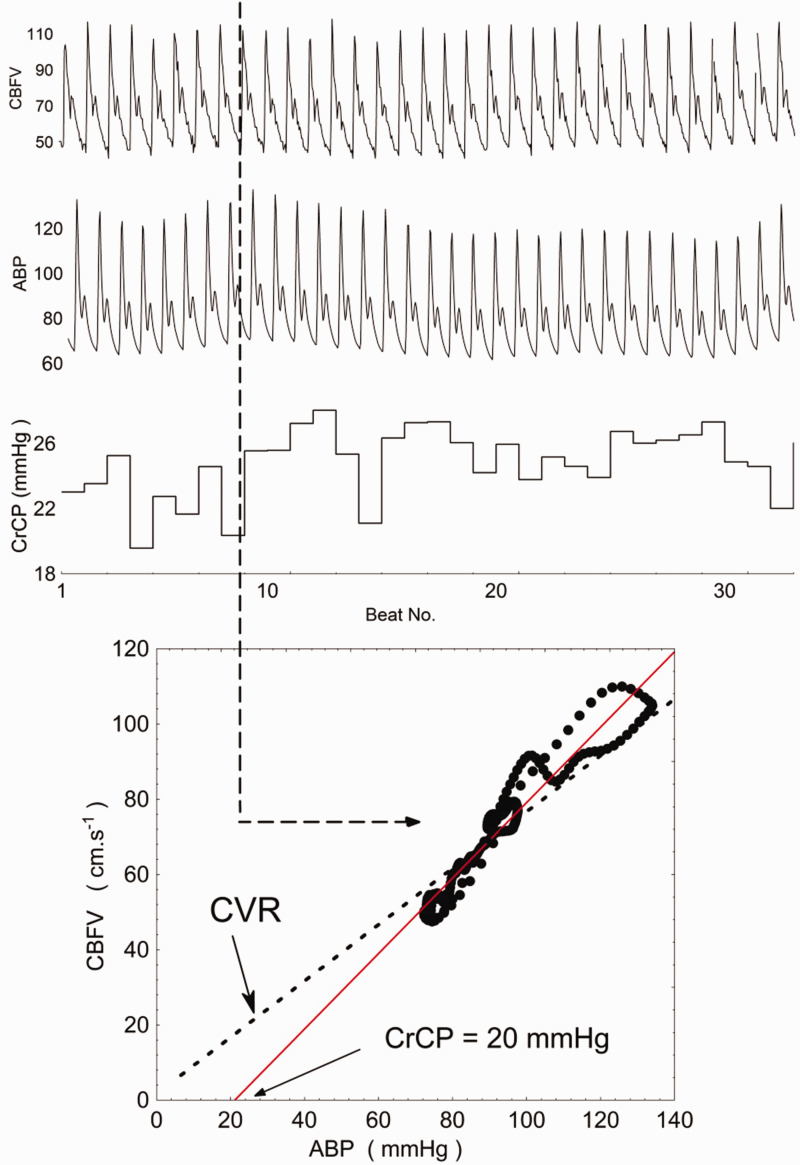
A demonstration of the differences in describing the BP-CBFv relationship between CVR and CrCP. Estimation of CrCP is calculated from CBFv and arterial BP recordings for a healthy subject, with an approximate CrCP of 20 mmHg. The dotted line illustrates how the relationship may be described using CVR, in which the relationship must cross the axes origin. (Modified from source: The critical closing pressure of the cerebral circulation, Medical Engineering & Physics^3^)

In view of the limitations of CVR, an alternative measure to assess CA is required. Reporting of CrCP and resistance-area product (RAP) parameters may instead be of value. Given the high temporal resolution of TCD, CrCP can be directly inferred from instantaneous CBv-BP curves for each cardiac cycle, with the gradient of the curves allowing estimation of RAP.^3^ The previously described flaw in the CVR concept is also highlighted by these CBv-BP curves by the absence of a line passing through the graph origin^4^ (Figure 1). Importantly, CrCP is not reserved to TCD measurements, but has also been reported with other flow measurement methods.^5^

In addition to being a more accurate representation of the CBF-BP relationship in the cerebral circulation, the use of CrCP and RAP has also been demonstrated to give clearer discrimination of cerebral haemodynamic changes than CVR alone.^4^ An important example is the vasomotor effect of arterial CO_2_, with hypercapnia causing vasodilation mainly by reduced values of CrCP,^3^ whilst the vasoconstriction induced by hypocapnia is predominantly caused by increased values of RAP.^6^

It has been hypothesised that CrCP could be expressing metabolic pathways of smooth muscle activation, whilst RAP could be reflecting myogenic responses to BP changes.^3,4,7^ It is also possible to speculate that the different sensitivity of CrCP and RAP to different stimuli could relate to segmental differences in vessel structure. For example, small arteries and large arterioles with a diameter that are less likely to collapse, may be more sensitive to myogenic control (reflected by RAP measurements), whereas small arterioles and capillaries controlled by the neurovascular unit via metabolic pathways could be affected more by the vascular waterfall mechanism, and having greater sensitivity to ICP changes, therefore being more expressed by CrCP.^1,2^

In a seminal communication, Burton demonstrated how CrCP is affected by the balance between transmural pressure and active wall tension.^2^ In the arterial cerebral circulation, transmural pressure is given by the difference between intra-arterial BP and the extra-vascular ICP, which has led to the suggestion that the difference mean BP-CrCP should be regarded as the ‘effective’ perfusion pressure of the cerebral circulation.^3,8–10^

Several reasons can be advanced to explain why the use of CVR in the literature remains entrenched, despite mounting evidence that it cannot express the true relationship between flow and pressure in the cerebral circulation. Firstly, estimation of CrCP and RAP involve an additional burden to investigators, requiring careful data editing and analysis to minimise the potential errors in their calculation. Next, the extrapolation of the instantaneous CBv-BP curve, to identify the point where CBv = 0, inevitably leads to greater susceptibility to noise and it is not unusual that CrCP will appear to be negative, a reason that has been put forward to dismiss its relevance. Furthermore, several methods have been proposed to estimate CrCP and RAP, resulting in a lack of consensus about which methods are most robust and reliable. Nevertheless, these practical limitations should not take precedence over the main point that the instantaneous CBF-BP relationship cannot be expressed by a single parameter, either CVR, or its inverse, the cerebrovascular conductance.

The debate generated by this *Point/Counterpoint* series should lead to improvements in scientific rigour and methodology. In studies of cerebral haemodynamics, replacing the use of the CVR parameter by the more accurate and informative combination of CrCP and RAP would be a step forward in that direction.
